# The Processing of Human Emotional Faces by Pet and Lab Dogs: Evidence for Lateralization and Experience Effects

**DOI:** 10.1371/journal.pone.0152393

**Published:** 2016-04-13

**Authors:** Anjuli L. A. Barber, Dania Randi, Corsin A. Müller, Ludwig Huber

**Affiliations:** Comparative Cognition, Messerli Research Institute, University of Veterinary Medicine Vienna, Medical University of Vienna and University of Vienna, Vienna, Austria; University of Lincoln, UNITED KINGDOM

## Abstract

From all non-human animals dogs are very likely the best decoders of human behavior. In addition to a high sensitivity to human attentive status and to ostensive cues, they are able to distinguish between individual human faces and even between human facial expressions. However, so far little is known about how they process human faces and to what extent this is influenced by experience. Here we present an eye-tracking study with dogs emanating from two different living environments and varying experience with humans: pet and lab dogs. The dogs were shown pictures of familiar and unfamiliar human faces expressing four different emotions. The results, extracted from several different eye-tracking measurements, revealed pronounced differences in the face processing of pet and lab dogs, thus indicating an influence of the amount of exposure to humans. In addition, there was some evidence for the influences of both, the familiarity and the emotional expression of the face, and strong evidence for a left gaze bias. These findings, together with recent evidence for the dog's ability to discriminate human facial expressions, indicate that dogs are sensitive to some emotions expressed in human faces.

## Introduction

An important perceptual-cognitive challenge for almost all animals is to find a compromise between 'lumping and splitting' when confronted with the vast amount of information arriving at their senses [1]. Animals need to categorize, i.e., to treat an object as belonging to a general class, but besides categorization of objects, they further need to recognize, i.e., to treat different images as depicting the same object, such as a specific face, despite changes in the viewing conditions. The brain of many animals solves both tasks in a natural, effortless manner and with an efficiency that is difficult to reproduce in computational models and artificial systems. Still, how this is accomplished is far from being fully understood.

An enormously rich source of information and an important category of visual stimuli for animals in all major vertebrate taxa is the face [2]. In many species faces convey, among other things, information about direction of attention, age, gender, attractiveness and current emotion of the individual (for reviews see [3–5]). Almost 50 years ago psychologists have demonstrated that facial expressions are reliably associated with certain emotional states [6,7]. This in turn can lead to behavioral decisions of a receiver concerning e.g. cooperation, competition, consolidation or formation of relationships. Some primates, for instance, seem to have evolved special abilities for reading faces due to their complex social lives (e.g. [8–10]).

While the categorization, interpretation and identification of faces from conspecifics is likely a consequence of the social life, which may have resulted in neural specialization for faces [11], it is a further challenge to achieve at least some of these abilities with faces of heterospecifics. The configuration of the face, the underlying facial muscles and the resulting expressions are more or less different from the own, depending on how taxonomically distant the other species is [12,13]. Nevertheless, a variety of animals have been shown to be able to identify and categorize faces and also emotions of heterospecifics (e.g. macaques [14], sheep [15], horses [16]). Several investigations on con- and heterospecific face processing suggest learned aspects in the information transfer and speed (c.f. face expertise, [17–22]). Hereby individuals are able to recognize and discriminate best between faces similar to those that are most often seen in the environment. For instance, the influence of individual experience with the other species was shown for urban living birds like magpies [23] and pigeons [24]. Especially early exposure to faces facilitates the ability of face discrimination [25,26]. Although some individuals are able to learn about heterospecific faces also later in life (e.g. chimpanzees [27], rhesus macaques [28]), they will not reach the same high level of competence [12,21]. It remains an open question, however, whether the improved abilities to read faces due to early life exposure are caused solely by an acquired early sensitivity for faces (innate mechanisms) or simply by the larger amount of experience (learned responses).

An ideal model to study such questions about experience for heterospecific faces is the domestic dog. Pet dogs live in an enduring intimate relationship with humans, often from puppy age on. As reviewed in several recent books [29–32], dogs have developed specific socio-cognitive capacities to communicate and form relationships with humans. Most likely caused by a mixture of phylogenetic (domestication) and ontogenetic (experience) factors, dogs living in the human household show high levels of attentiveness towards human behavior (reviewed in [33]), follow human gestures like no other animal, (e.g. [34]), exhibit a high sensitivity to human ostensive signals, like eye contact, name calling and specific intonation [35] and show an increased readiness to look at the human face [36]. In comparison to hand-reared wolf puppies, the latter is already present in dog puppies ([37]; see also [38]). By monitoring human faces, dogs seem to obtain a continuous stream of social information ranging from communicative gestures to emotional and attentive states. Even if this does not mean that dogs are readers of our minds but only exquisite readers of our behavior [39], they evaluate humans on the basis of direct experiences [40] and are sensitive to what humans can see (a form of perspective-taking; reviewed by Bräuer [41]).

A special sensitivity for faces in dogs is further supported by two recent findings. An fMRI study in awake dogs has revealed that a region in the temporal cortex is face selective (it responds to faces of humans and dogs but not to every-day objects, [42]). Dogs also have been found to show a gaze bias towards faces [43,44]. Still, there is mixed evidence for the lateralization towards conspecifics and heterospecifics. Dogs showed a human-like left gaze bias toward human faces but not toward monkey or dog faces [44]. This left gaze bias was exhibited only toward negative and neutral expressions, however, but not toward positive expressions of human faces [43]. It has been proposed that the right hemisphere is responsible for the expression of emotions and that the assessment of emotions therefore leads to a left gaze bias towards such stimuli (right hemisphere model, [45,46]). In contrast to the right hemisphere model, the valence model [47,48] suggests that the left hemisphere mainly processes positive emotions while the right hemisphere mainly processes negative emotions. So far a variety of species (e.g. humans[49,50], apes [51], monkeys [52], sheep [53], dolphins [54], dogs [43,55–57], etc.) have been shown to show lateralization towards emotive stimuli (reviewed by Adolphs [58] and Salva [59]).

The dog's following of the human gaze and, more generally, the visual scanning of faces require the application of advanced technical tools. The most commonly used equipment in human psychophysical laboratories is the eye-tracker [60,61]. Besides the gaze-following study by Téglás and colleagues [62], two studies have so far used this technology for the investigation of the looking behavior of dogs. In a kind of proof-of-concept study, Somppi and colleagues [63] showed that dogs are looking with a higher frequency to the informative parts of a picture and that they direct more attention to conspecifics and humans compared to objects. In a follow-up study addressing the looking at faces, the researchers could demonstrate that dogs pay more attention to the eye region compared to the rest of the face [64]. The finding that the mean fixations were longer for conspecific compared to heterospecific (human) faces suggests that dogs process faces in a species-specific manner. The use of the eye-tracker revealed subtle differences in looking patterns between dogs living in different human environments: pet dogs and kennel dogs. Still, both types of dogs showed a preference for the own species, familiar pictures and the eye region. Overall, these findings suggest that the living conditions of dogs affect the way they look at us. However, it is not clear whether this is due to the limited time in close relationship with humans or to the development of human face processing during early life.

This eye-tracking study addresses this question by comparing the looking at human faces between pet and lab dogs. For both types of dogs the human environment during their whole life is known, especially the difference in the amount of exposure to humans and the quality of the human-dog relationship. A further way to investigate the role of individual experience with human faces is to compare the dogs' looking at familiar and unfamiliar faces. It is already known that dogs are able to discriminate between familiar and unfamiliar faces, either implicitly by exhibiting looking preferences [65,66] or by making the discrimination explicit in a two-choice task [67]. However, it is not yet known if dogs show preferences and respond to differences between familiar and unfamiliar faces only if forced to do so or if dogs spontaneously scan familiar faces and unfamiliar faces differently.

A further interesting but not yet investigated influence on the dog's looking pattern at human faces is the facial expression as a result of the human's emotion. Recently we could show—confirming and extending the findings of Nagasawa and colleagues [68]–that dogs can learn to distinguish between two facial expressions of the same unfamiliar human persons and to generalize this ability to novel faces [69]. Importantly, our study suggested this ability is not dependent on the exploitation of simple features like the visibility of teeth but rather on the memory of how different human facial expressions look like. These memories are likely being formed in everyday interactions with humans, especially with the dogs' human partner(s).

Therefore, in addition to possible effects of experience (pet dogs vs. lab dogs, familiar faces vs. unfamiliar faces), this study focusses on the question on how dogs process emotional faces (angry, happy, sad, and neutral). In line with a previous eye-tracking study [64], we expected that lab dogs, due to their limited experience with humans, would show longer latencies, shorter fixation durations and a tendency to fixate more on familiar faces compared to pet dogs. Further we hypothesized that there is a difference between the processing of a familiar and unfamiliar face as the emotional expressions of a familiar face should be processed faster due to previous experience with the face. Based on the findings of our recent study [69], we expected that the four emotional expressions are processed differently and elicit a different amount of attention (i.e. differing amount of fixations and fixation durations). Additionally, in this previous study dogs required more training to discriminate happy and angry faces if they had to touch the angry face. This is in contrast to dogs that had to touch the happy face. The results indicate that they avoided approaching the angry faces. Therefore we expected that they fixate faces that are expressing negative emotions shorter and less often. Concerning the scanning pattern, we expected that more informative parts of the face (eye and mouth region) are fixated more frequently. Finally, we expected the dogs to show a gaze bias towards the faces.

### Methods

All experimental procedures were approved in accordance with GPS guidelines and national legislation by the Ethical Committee for the use of animals in experiments at the University of Veterinary Medicine Vienna (Ref: 09/08/97/2012). All dog owners gave written consent to participate in the study. The individual in the figures of this manuscript has given written informed consent (as outlined in PLOS consent form) to publish these case details.

### Subjects

Nineteen privately owned pet dogs and eight laboratory dogs participated in the study (see Table 1). The pet dogs were of various breeds and their age ranged between 1–12 years. Dog owners of the pet dogs spent on average 27 h/week actively with their dogs (walking, playing and training). With the exception of two dogs, all dogs were at least one to two times a week active in dog sports activities like agility, dog dance, assistant dog training, man trailing etc. The two remaining dogs had only training as a “Begleithund” and obedience training on a daily basis by their owners. Their owners gave written consent to participate in the study. The laboratory dogs were all beagles, aged 1–5 years, and were housed on the campus of the University of Veterinary Medicine Vienna in packs of either 13 (Clinical Unit of Internal Medicine Small Mammals (CU-IM), L1-L6 in Table 1) or 4 dogs (Clinical Unit of Obstetrics, Gynecology and Andrology (CU-OGA), L7 and L8 in Table 1). None of the laboratory dogs ever had done any activity in dogs sport or ever participated in professional obedience training. They were born at the Clinical Unit of Obstetrics, Gynecology and Andrology at the University of Veterinary Medicine Vienna. After weaning off from the mother at the age of two month, the dogs were living in packs. The housing of both groups consisted of an indoor and outdoor enclosure (CU-IM: indoor enclosure = 64qm, outdoor enclosure = 248qm; CU-OGA:indoor enclosure = 18qm, 3 outdoor enclosures = ~70qm), in which they could move freely during the day. From 22:00–6:00 o´clock the dogs were kept in the indoor enclosures. The enclosures were enriched with dog toys (chewing, squeaking toys or balls). Their contact to humans was limited to the daily feeding (once a day in the morning, water ad libitum) and cleaning of the enclosures of the animal keepers. Additionally they were participating in practical courses for the training of students of veterinary medicine (approx. 1–2 times a semester). However, none of the dogs was socialized in a classical way, as they were living only with their conspecifics, but not with humans.

**Table 1:** List of participating dogs.

Dog ID^(^^a^^)^	Breed	Age^(^^b^^)^	Sex^(^^c^^)^	Status	Valid Sessions	Valid Trials
P1	Australian Kelpie	1	m	neutered	4	59
P2	Australian Shepherd	1	m	intact	-	-
P3	Australian Shepherd	2	f	neutered	2	14
P4	Australian Shepherd	3	m	intact	5	56
P5	Bernese Mountain Dog	2	f	neutered	2	27
P6	Berger Blanc Suisse	5	m	intact	2	15
P7	Border Collie	1	m	intact	5	76
P8	Border Collie	6	m	neutered	4	60
P9	Border Collie	8	m	neutered	5	57
P10	German Shepherd	6	f	neutered	4	68
P11	Hungarian Viszla	3	m	neutered	5	60
P12	Irish Wolfshound	1	f	intact	3	31
P13	Mongrel (American Bulldog-Mix)	11	m	neutered	3	37
P14	Mongrel (Beauceron-Husky-Mix)	3	m	neutered	4	61
P15	Mongrel (Bracke-Shepherd-Mix)	8	m	neutered	4	42
P16	Mongrel (Mudi-Schnauzer-Mix)	2	f	neutered	5	65
P17	Mongrel (Spitz-Peke-Mix)	11	m	neutered	-	-
P18	Standard Poodle	6	m	intact	3	51
P19	Puli	1	f	intact	5	68
L1	Beagle	3	m	neutered	4	62
L2	Beagle	4	m	neutered	5	70
L3	Beagle	1	m	neutered	5	75
L4	Beagle	4	m	neutered	5	57
L5	Beagle	1	m	neutered	5	69
L6	Beagle	3	m	neutered	5	64
L7	Beagle	1	m	intact	5	69
L8	Beagle	1	m	intact	4	51

^a^ P = pet dog, L = lab dog.

^b^ Age in years.

^c^ m = male, f = female.

### Experimental setup

**Fig 1:**
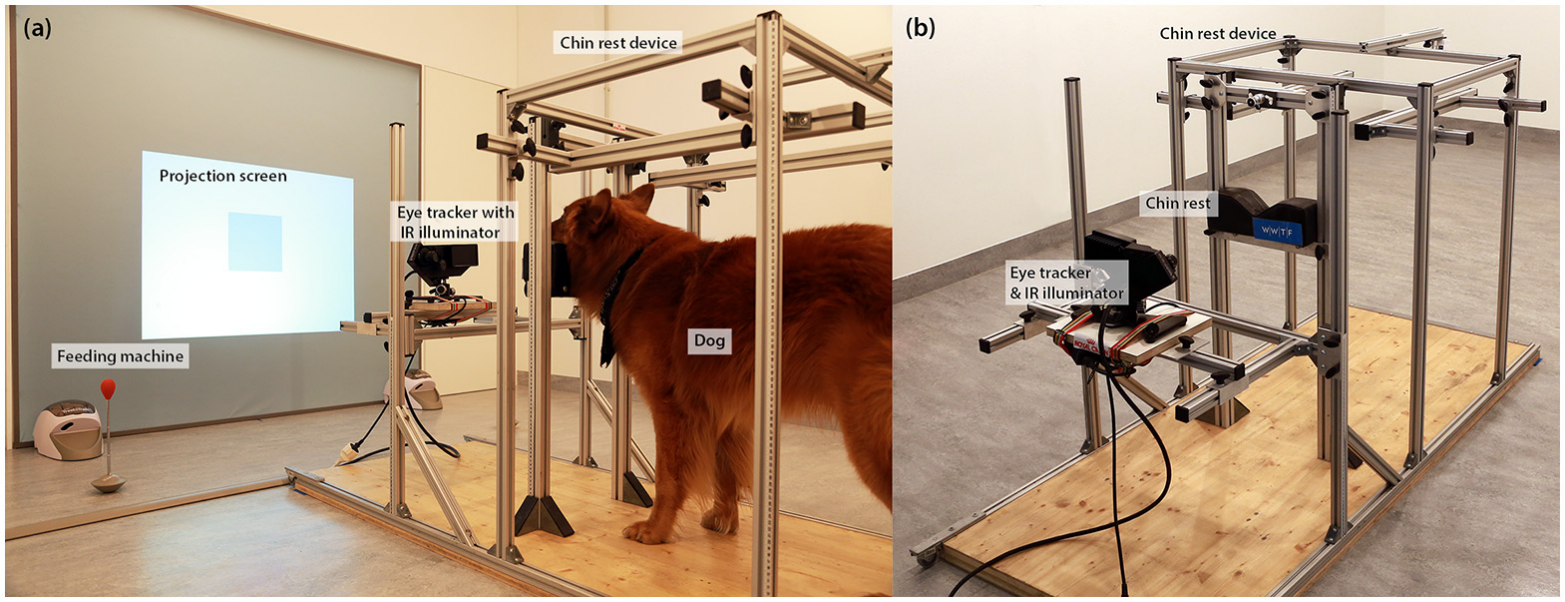
Experimental set up. (a) Lateral view of chin rest device, projection screen and feeding machines seen from the rear of the room during training with geometrical figures and (b) front view of the chin rest device including the eye-tracking camera with IR illuminator.

All experiments were conducted at the Clever Dog Lab of the University of Veterinary Medicine, Vienna. The experimental room was divided by a wall, consisting of a projection screen (200 x 200 cm) and two doors, into two compartments; a small one (149 x 356 cm) housing the computer system operating the eye tracker and a video projector (NEC M300XS, NEC Display Solutions, United States), and a larger one (588 x 356 cm) with the chin rest device and the eye tracker (see Fig 1). The stimuli were back-projected onto the projection screen (size projection area 110 x 80 cm). We used the eye tracking system Eyelink 1000 (SR Research, Ontario, Canada) to record monocular data from the subjects. This system is ideal for dog research because it can be used with a chin rest or without any head support and with a remote camera if head fixation is not desirable, but high accuracy and resolution are still important. We used a customized chin-rest device for head stabilization. A pillow with a v-shaped depression was mounted on a frame to allow vertical adjustment of the chin rest to the height of the individual dog. The frame consisted of aluminium profiles (MayTec Aluminium Systemtechnik GmbH, Germany) that allowed the easily adjustable but stable fixation of additional equipment (e.g. cameras). The chin rest was positioned at a distance of 200 cm from the projection screen. The eye-tracking camera with the infrared illuminator was mounted on an extension of the chin-rest frame (see Fig 1). This apparatus was aligned horizontally with the chin rest at a distance of 50 cm. Light conditions in the room were kept constantly at 75 lux using LED-light bulbs (9,5W, 2700k, Philips GmbH Market DACH, Germany).

### Stimuli

**Fig 2:**
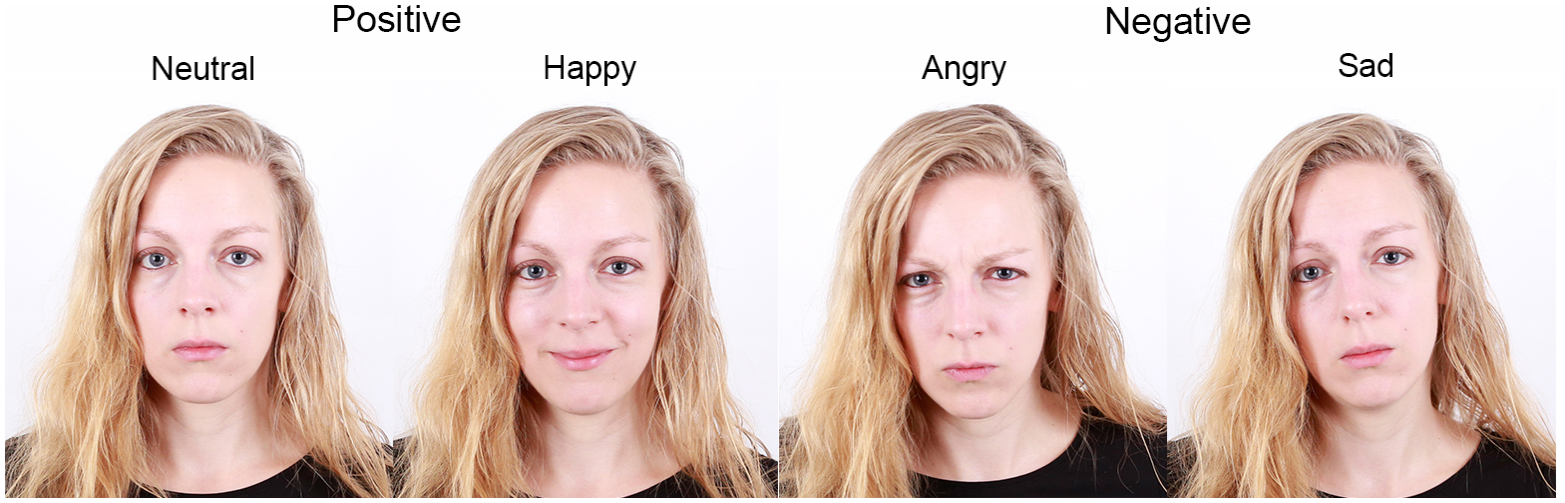
Examples of the experimental stimuli.

The stimulus set for each subject consisted of photographs of the face of the dogs' owner (for the lab dogs the dog trainer, who trained them for the experimental tests) and of an unfamiliar person expressing four different emotions: neutral, happy, angry and sad (Fig 2). All owners and all unfamiliar humans were female. The photographs were taken by experimenter 1 (E1) in a standardized setup at the Clever Dog Lab in front of a white background. For each dog the faces of the owner served as familiar stimuli and the faces of another dog´s owner participating in the study were used as unfamiliar stimuli. For the unfamiliar stimuli, a person was chosen who did not differ strongly from the owner in appearance (e.g. hair color). The stimuli were projected onto the screen with 1024 x 768 px resolution, resulting in 900 x 850 mm sized pictures which corresponds to 25.36° x 23.99° of visual angle (head: approx. 400 x 300 mm corresponding to11.42° x 8.58° of visual angle).

The stimuli were validated concerning their emotional expression and valence afterwards by 67 neutral observers via an online-questionnaire. All participants were recruited via the internet (17 male, 50 female, age range 16–55 years). The participants were asked for every picture to rate the emotion angry, happy, neutral and sad on scale from 0–100% (e.g. Picture xy = 0% angry, 0% happy, 30% neutral, 70% sad, the four numbers had to add to 100%). For every picture data of all ratings for the certain emotions were pooled and it was decided if the picture was identified correctly. Identification of the correct emotion was given when rated as the predominantly shown emotion, higher at least 15% than the second highest rated emotion, e.g. if an angry picture was rated with 62% angry, 0% happy, 23% neutral and 13% sad it was considered as correctly identified. In contrast, if the same picture was rated with 52% angry, 0% happy, 38% neutral and 10% sad would not have been considered as correctly identified. Validation revealed that for the angry condition 74%, happy 95%, neutral 100% and sad 60% of the pictures were identified correctly. The negative emotions were usually mistaken with a neutral expression (angry in 80% of the cases, sad in 88% of the cases). Incorrect rated pictures were left in the analysis as we wanted the dog owners to express emotional expressions as natural as possible. This resulted in expressions which could be ambiguous for non-informed observers.

### Procedure

#### Training

Prior to the experiment, the dogs were trained for the experimental procedure (for 2–6 months). Training was done by E1 and E2. We used operant conditioning with a positive reinforcement (food rewards) and a clicker as a secondary reinforcer. The dogs were trained once or twice a week, depending on their availability. The owner of the dog was allowed to stay in the room during the whole procedure. She was positioned on a chair at the back of the room and instructed to avoid interaction with the dog.

The training consisted of three steps. In the first step the dog was trained to go to the chin rest device and to rest the head on the chin rest for at least 10 sec (see Fig 1a). After the dog had learned to stay in the chin rest it was confronted with a two-choice conditional discrimination task with geometrical figures (GFs). With this task we aimed to increase the dog’s attention to the screen (up to 20 sec) and to make the training more interactive. Furthermore, this step was included to prepare the dog for the calibration procedure during which the dog had to follow the position of 3 dots on the screen (see below). In the last training step, pictures of landscapes, animals, architecture and humans (but not of human faces expressing emotions) were interspersed between the GFs to habituate the dog to the presentation of pictures and to increase the duration the dog could leave its head motionless on the chin rest further. These pictures varied in size between 50 x 50 and 100 x 100 cm. Additionally we introduced the presentation of small animated videos with sound (interspersed within the blocks of pictures) which were used as attention triggers during the test phase. Training was considered successful if the dog, in 4 out of 5 trials, stayed motionless on the chin rest with their head and oriented towards the screen for at least 30 seconds while the stimuli were presented. Orientation was determined by the experimenter via visual inspection aided by the on-the-fly output of the eye tracker.

#### Testing

Testing was done by AB and DR. During the whole procedure the owner was allowed to stay at the back of the room and was instructed to avoid interaction with the dog. At the beginning of a test session, the dog was allowed to explore the room for approximately 5 min. Following that, the eye tracking system was calibrated with a three point calibration procedure. Thereby three small dots were presented one after the other on the screen (size of the dot: 50 mm, coordinates (x,y): (512,65), (962,702), (61,702)) while the dog had its head on the chin rest. When the dog focused on the dot for at least a second, the point was accepted by E1 on the operating computer. After calibration of the three points, a validation of the accepted calibration points followed. For this purpose all three calibration points were shown again. If the validation revealed an average deviation of more than 3° of visual angle between the two repetitions, the calibration was rejected and repeated until this criterion was met. Calibration was done before every session. Following successful calibration, the dog was presented with the stimuli as follows:

1. Before the presentation of each picture, an attention trigger (small animated video) was presented on the screen. The position of the trigger was randomly assigned to one of the four corners of the screen (x,y: (150,150), (150,620), (875,150), (875,620)). The size of the trigger was 5 cm on the screen which corresponds to 1.43 degrees of visual angle. The presentation software of the eye tracker (Experimental Builder 1.10.1241, SR Research Ltd., Ontario, Canada) was programmed so that presentation of the stimuli followed immediately and automatically once the dog was fixating on the trigger.
2. Faces were presented for five seconds in the center of the screen (x,y: (512,382)).
3. Faces were presented in **blocks** of four trials (each trial consisted of the presentation of a trigger followed by the presentation of a face picture). The blocks contained pictures of either only positive (happy and neutral) or only negative (angry and sad) facial expressions. The order of the presentation was randomized within the block, but every block contained both corresponding emotional expressions (either positive or negative) of the familiar and the unfamiliar person.
  After the end of each block, the dog received a food reward. Depending on the dog´s motivation and concentration a short pause (up to 1 min) was made before the dog was asked to reposition itself in the chin rest device. During this pause the dog was allowed to rest or to move freely in the room.
4. Within one **session** four blocks of pictures were presented. Two blocks contained positive and two blocks contained negative facial expressions. Each of the eight pictures of a dog’s stimulus set was shown twice in each session.

Every dog completed five sessions (one session per week) with the exception of three dogs for which we discontinued data acquisition due to a lack of motivation (for example if the dog did not want to go back to the chin rest anymore or did not stay in the chin rest during picture presentation). For this reason data acquisition with one dog was stopped after two sessions and for two other dogs after four sessions. The available data of these dogs was nevertheless included in the analyses (see data-preparation).

### Data analysis

To be included in the analyses, data had to fulfil following criteria: Trials were only included in the analysis if the dog fixated at least once into the face. Additionally, a session was only retained if the dog had fixated at least once on a face of both emotional conditions (positive and negative). The data of a dog were discarded completely if it did not include at least two valid sessions (for further details see Table 1).

**Fig 3:**
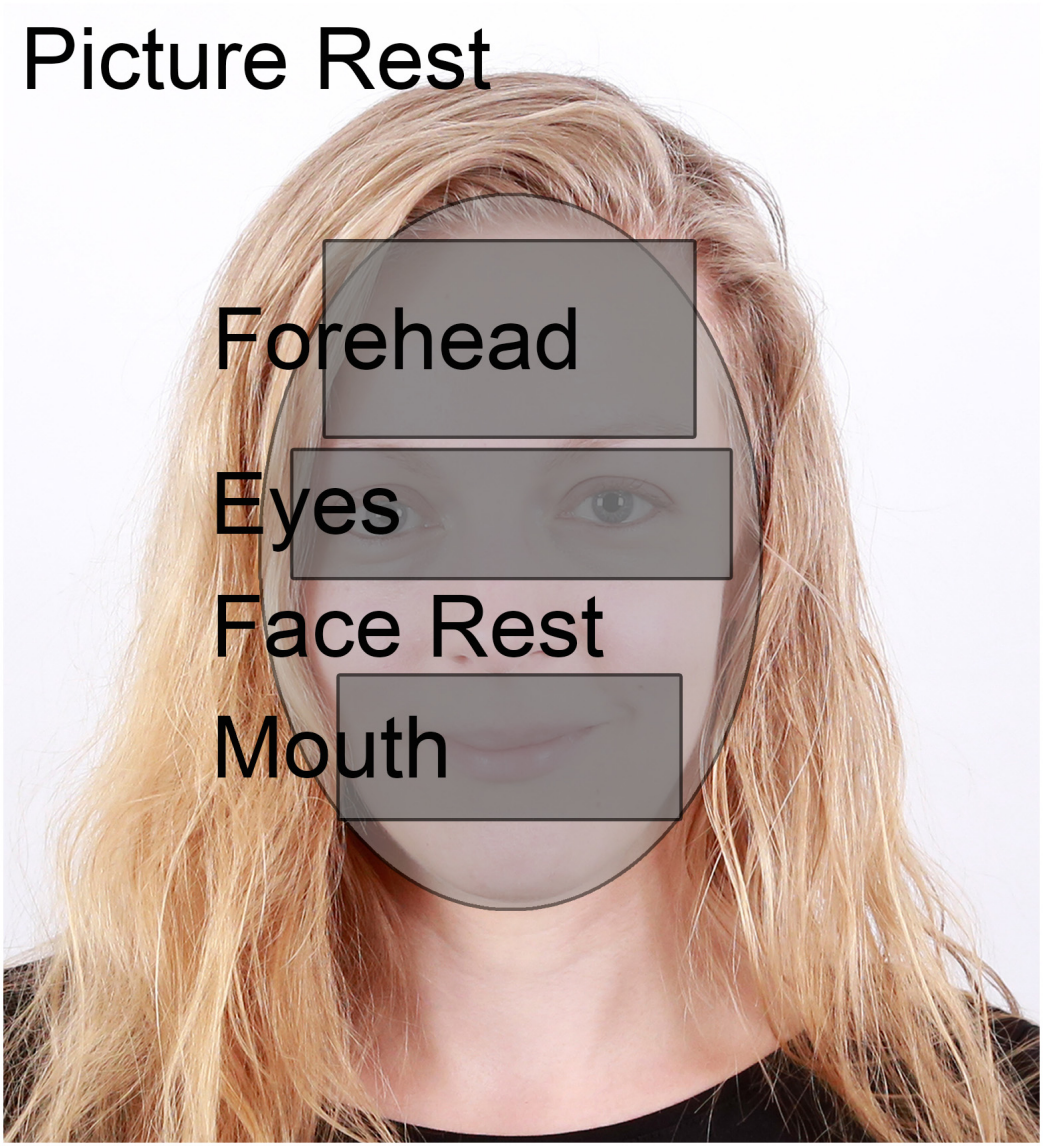
Scheme showing the areas of interest (AoI) for forehead, eyes, face rest, mouth and rest of the picture.

Each face was divided into five areas of interest (AoI): eyes, forehead, mouth, face rest (nose, cheeks and chin) and picture rest (hair and background, see Fig 3). Size and positioning of the AoIs was based on the following rules:

1. AoI forehead: The forehead area was a rectangular area ranging upwards from the top of the eyebrows to the highest point of the hairline bounded by the face contours to the left and the right.
2. AoI eyes: The eye area ranged from the middle of the pupil to the eyebrow. This area was mirrored downwards and bounded by the face contours to the left and the right.
3. AoI mouth: The mouth region ranged from the middle of the mouth to the base of the nose. This area was mirrored downwards and bounded by the face contours to the left and the right.
4. Face rest: Included all parts of the face area not belonging to AoI (1)-(3).
5. Picture rest included all remaining parts of the picture that did not belong to AoI (1)-(4) (e.g. blank parts, hair, shoulder etc.)

Fixations into the AoIs (1)-(4) are summarized as “in-face” fixations. Raw eye movement data was analysed using Data Viewer 2.1.1 (SR Research, Ontario, Canada). Eye movement events were identified by the EyeLink tracker´s on-line parser. As there is to date no validated literature on the definition of eye movement events in dogs, we were working with raw data without any thresholds for fixation duration. From the raw data we extracted fixation durations, number of fixations and latencies to the first fixation (fixation start). Note that fixation durations were not trimmed and durations could therefore exceed the presentation time of a trial if a dog was holding a fixation longer than the picture presentation. On the basis of these data we calculated mean values for fixation durations and number of fixations over each trial. As we were interested in face processing (rather than picture processing) only fixations into the face region were included in the analyses unless state otherwise. For this reason, we extracted the fixation duration of the first fixation directed into one of the four in-face AoIs, the number of fixations directed into in-face AoIs, the identity of the AoI of the first in-face fixation, the vector (angle) between the first and second fixation (gaze direction) and the latency of the first fixation into one of the in-face AoIs.

Statistical analyses were conducted with SPSS 22 (IBM Corp., Armonk, New York, United States) and R 3.1.1 [70]. Linear mixed effect models (LMM) were used to analyse mean fixation duration, fixation duration of the first fixation, overall fixation count per trial and the latency to the first fixation (time). As fixation counts into the four different AoIs did not fulfill the assumptions of a Poisson distribution (in particular zeros were strongly overrepresented), we used zero-inflated negative binomial models to analyze these data (number of fixations into the different AoIs), using the R-package glmmADMB 0.8.0 [71]. Likewise, the fixation number of the first fixation into one of the four in-face AoIs was analysed (latency of first fixation into in-face AoI, note that the first fixation was thereby labelled as zero). Generalized linear mixed models (GLMMs) were used to analyse if the first fixation was directed into the face region (SPSS, binomial GLMM) and which AoI was fixated first (first AoI, SPSS, multinomial GLMM). Using the R-package lme4 1.1.7 [72], we analysed if the first fixation was directed to the left or right half of the face (binomial, GLMM). Additionally we calculated the proportion of the fixation time dwelled on the left half of the face ((average duration left*100)/(average duration left-average duration right), negative binomial GLMM).

We could not find any difference between the emotions angry and sad as well as happy and neutral. For this reason, and to increase statistical power, the “positive” emotional expressions, happy and neutral, and the “negative” emotional expressions, angry and sad, were pooled. All linear models included emotion type (positive vs. negative), familiarity (familiar vs. unfamiliar), dog type (pet vs. lab dog) and where applicable AoI (eye, forehead, mouth, face rest), as well as all 2-way interactions between them, as fixed effects. Where 2-way interactions turned out significant, the dataset was split to explore the nature of the interaction. Dog type and session number nested within dog type were included as random factors in all models. Additionally we tested if the trial number (nested within session) and the age of the dog could explain a significant proportion of the variation in the models by adding these variables as random factors. Both variables did not have a significant influence on the data (decision on basis of Akaike´s information criterion (AIC)). We also tested for an effect of breed on the data. For this purpose, we combined data of dogs that are known to belong to the group of herding (P1-5, P7-9, P14, P19), hunting (P11, P12, P18, P15, L1-8) or protection dogs (P5, P10, P13; categorization on basis of their use as working dog). We compared the group of laboratory beagles (hunting dogs) to the three breed groups of the pet dogs. We did not find significant effect concerning the breed group. This factor was discharged from further analysis. Model reduction was done backwards on the basis of Akaike´s information criterion (AIC). Predictors were assumed to be significant with α ≤ 0.05.

## Results

The final dataset included data from 17 pet dogs (11 male and 6 female) with a total of 66 sessions and 847 trials (percentage trials with positive emotion 48.4%, mean 50 trials/dog, ranging from 14 to 76) and 8 lab dogs with a total of 38 sessions and 517 trials (percentage trials with positive emotion 50.7%, mean 65 trials/dog, ranging 51 to 74).

### Fixation Duration

#### Total fixation time per trial

**Fig 4:**
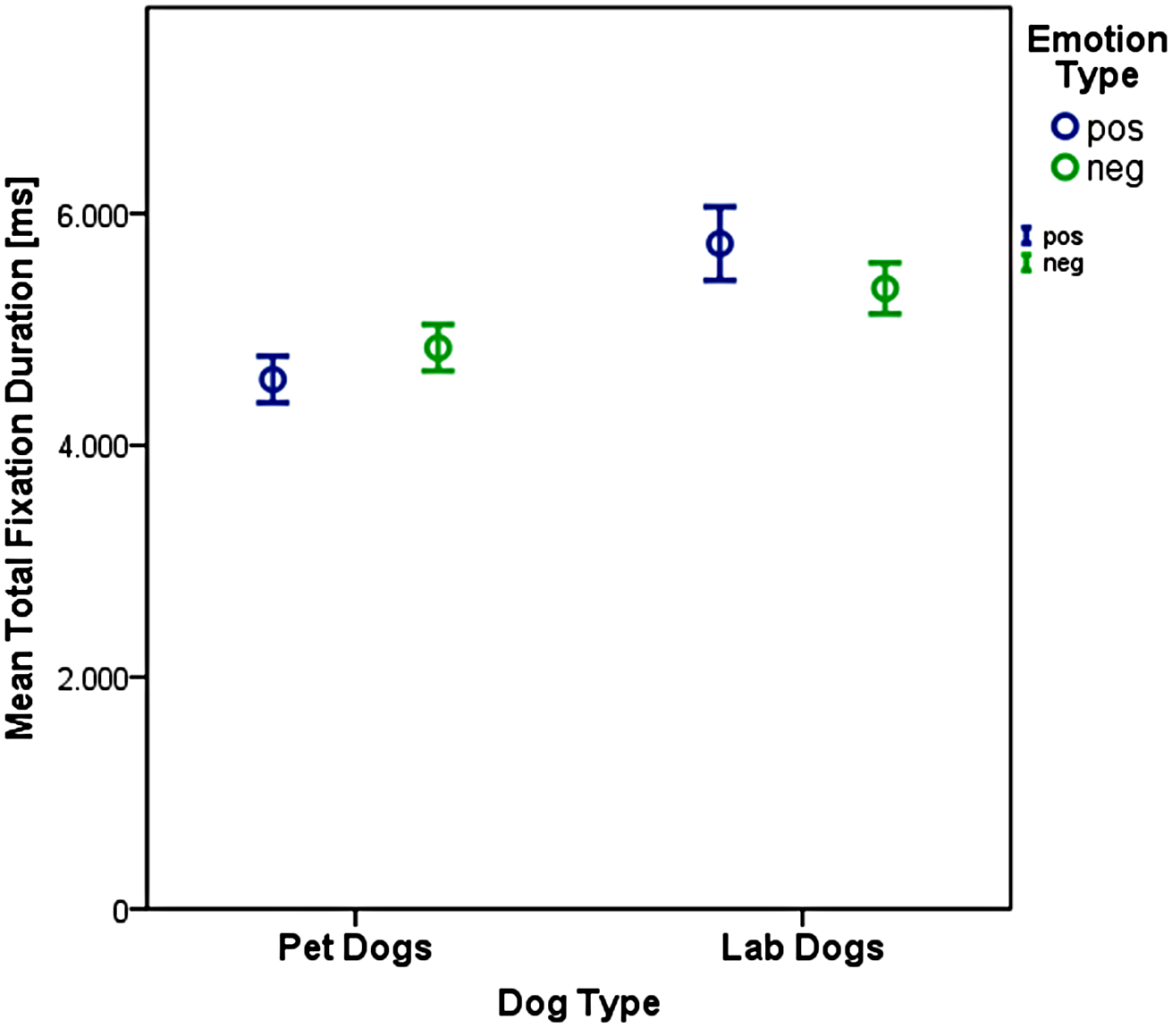
Total fixation time (+/- 95% CI) per trial for pet and lab dogs and subdivided for the emotion type.

There was a significant difference between pet and lab dogs concerning the total time they spent fixating the picture (LMM: F_1,19.6_ = 7.27, p = 0.014). The lab dogs spent more time fixating the picture (5545 +/- 98 ms) than the pet dog (4701 +/- 72ms, see Fig 4). Also there was a significant interaction between dog type and emotion type (F_1,1271.2_ = 6.15, p = 0.013). The pet dogs looked longer at the negative compared to the positive emotions (F = _1,795.1_ = 1.16, p = 0.28) whereas the lab dogs spent more time looking at the positive compared to the negative emotions (F_1, 473.6_ = 4.35, p = 0.04, see Fig 4).

#### Average fixation duration per fixation

**Fig 5:**
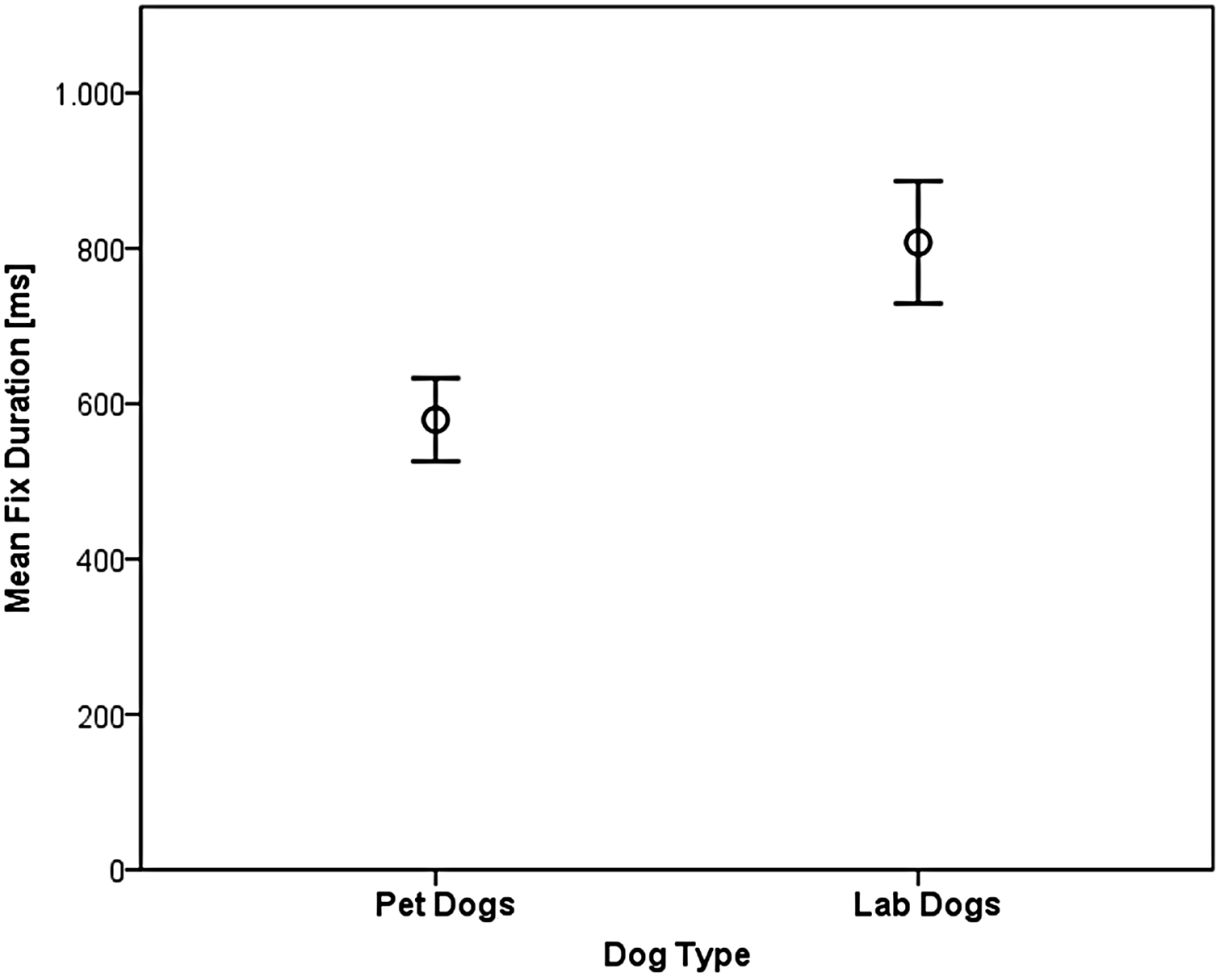
Average fixation duration (+/- 95% CI) of the first fixation directed into the face region for pet and lab dogs.

The dogs fixated the picture on average for 827 ms +/- SE 16ms. Average values did not differ between the pet (807 +/- 19ms) and lab dogs (858 +/- 28ms, LMM: F_1,21.4_ = 0.29, p = 0.59, see Table 1). The average fixation duration was not influenced by the emotion type (F_1,1535,3_ = 0.36, p = 0.55) or the familiarity of the face (F_1,1529.5_ = 1.55, p = 0.21). However, analysis of the fixation duration of the first fixation directed into the face revealed a significant difference between the two dog types: the first fixations of the lab dog (822+/- 40ms) were significantly longer than the first fixations of the pet dogs (582+/- 27ms, LMM: F_1,19.7_ = 4.55, p = 0.046, see Fig 5). We found no influence of emotion type (F_1,668.5_ = 0.98, p = 0.32), familiarity (F_1,651.6_ = 0.83, p = 0.36) or area of interest fixated (F_3,685.1_ = 0.91, p = 0.44) on the duration of the first fixation.

### Fixation Count

The mean number of fixations made during a trial (including fixations not directed into the face) was 8.07 +/- SE 0.14. This number did not differ between pet dogs (7.86 +/- 0.19) and lab dogs (8.43 +/- 0.23; LMM F_1,21.2_ = 0.65, p = 0.43). Also, there was no influence of emotion type (F_1,324.9_ = 0.16, p = 0.69) or familiarity (F_1,1075.4_ = 0.42, p = 0.52) on the mean number of fixations.

Less than half of all fixations directed to the picture were made into the face region (total = ~8, in-face = ~3, see Table 2). Still, there was a significant difference concerning the number of fixations made into the areas of interest during a trial (GLMM Likelihood Ratio Test (LRT) Chi²_3_ = 141.8, p< 0.001). The face rest region was fixated on average once per trial whereas the forehead region was fixated on average just half as often (c.f. Table 2). Eyes and mouth regions received on average the same number of fixations per trial (c.f. Table 2).

**Table 2:** Mean values of the fixation count into the areas of interest per trial.

Area of interest, Sum +/- SE	Pet (N = 847)	Lab (N = 517)	Positive (N = 692)	Negative (N = 672)
Face region	3.4 +/- 0.18	2.6 +/- 0.16	3.1 +/- 0.18	3.1 +/- 0.19
Eye	0.79 +/- 0.046	0.65 +/- 0.038	0.73 +/- 0.044	0.74 +/- 0.047
Mouth	1.07 +/- 0.058	0.4 +/- 0.034	0.73 +/- 0.048	0.91 +/- 0.062
Forehead	0.49 +/- 0.032	0.6 +/- 0.036	0.61 +/- 0.037	0.45 +/- 0.031
Face rest	1.05 +/- 0.047	0.98 +/- 0.048	1.05 +/- 0.050	1.00 +/- 0.047

**Fig 6:**
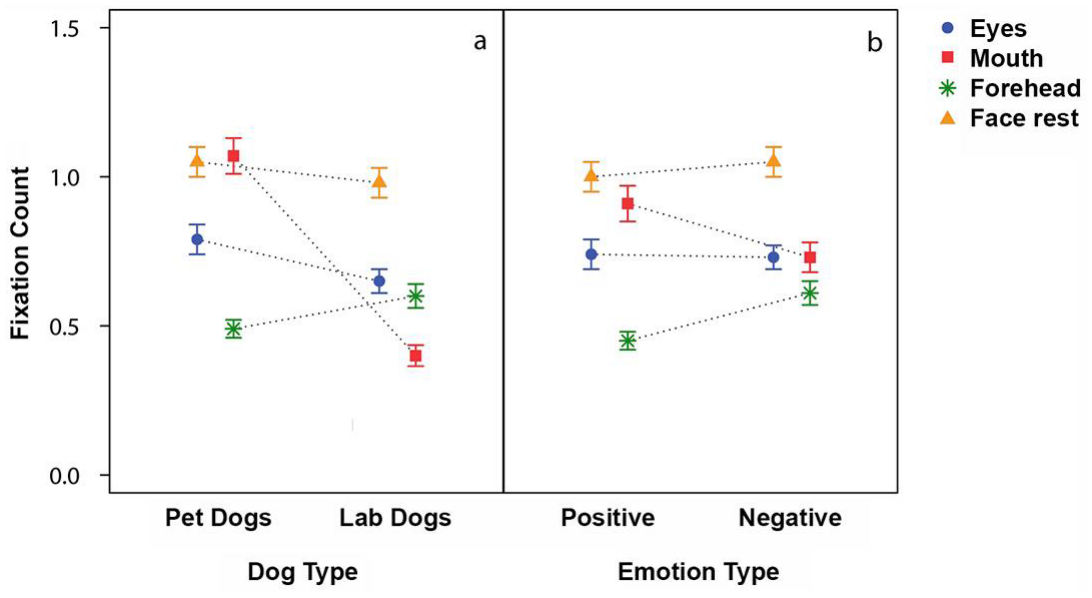
Fixation count (Mean+/-SE) into an area of interest during a trial subdivided by dog type (a) and emotion type (b).

There was a significant interaction between AoI and dog type (LRT Chi²_3_ = 100.2, p<0.001). The mean fixation count of pet and lab dogs differed significantly for the mouth region (estimate = -0.8 +/- 0.32, z = -2.53, p = 0.011) but not for the eyes (GLMM estimate = -0.08 +/- 0.22, z = -0.36, p = 0.72), the forehead (estimate = 0.42 +/- 0.26, z = 1.61, p = 0.11) or the face (estimate = -0.02 +/- 0.14, z = -0.17, p = 0.86). On average, the pet dogs looked to the mouth region about once per trial (mean +/- SE; 0.82 +/- 0.04), whereas the lab dogs fixated the mouth region less than half as often (0.4 +/- 0.03; see Fig 6). There was a significant interaction between AoI and emotion type (LRT Chi²_3_ = 23.1, p<0.001). The forehead was fixated significantly more often when a positive expression was shown compared to a negative expression (estimate = 0.25 +/- 0.08, z = 3.0, p = 0.003) whereas the mouth was fixated significantly more often when a negative expression was shown compared to a positive expression (estimate = -0.18 +/- 0.08, z = -2.29, p = 0.02, see Fig 6). The fixation count to the other two AoIs (eyes, face) did not differ significantly between the two types of emotion.

### Area of interest of the first fixation

**Fig 7:**
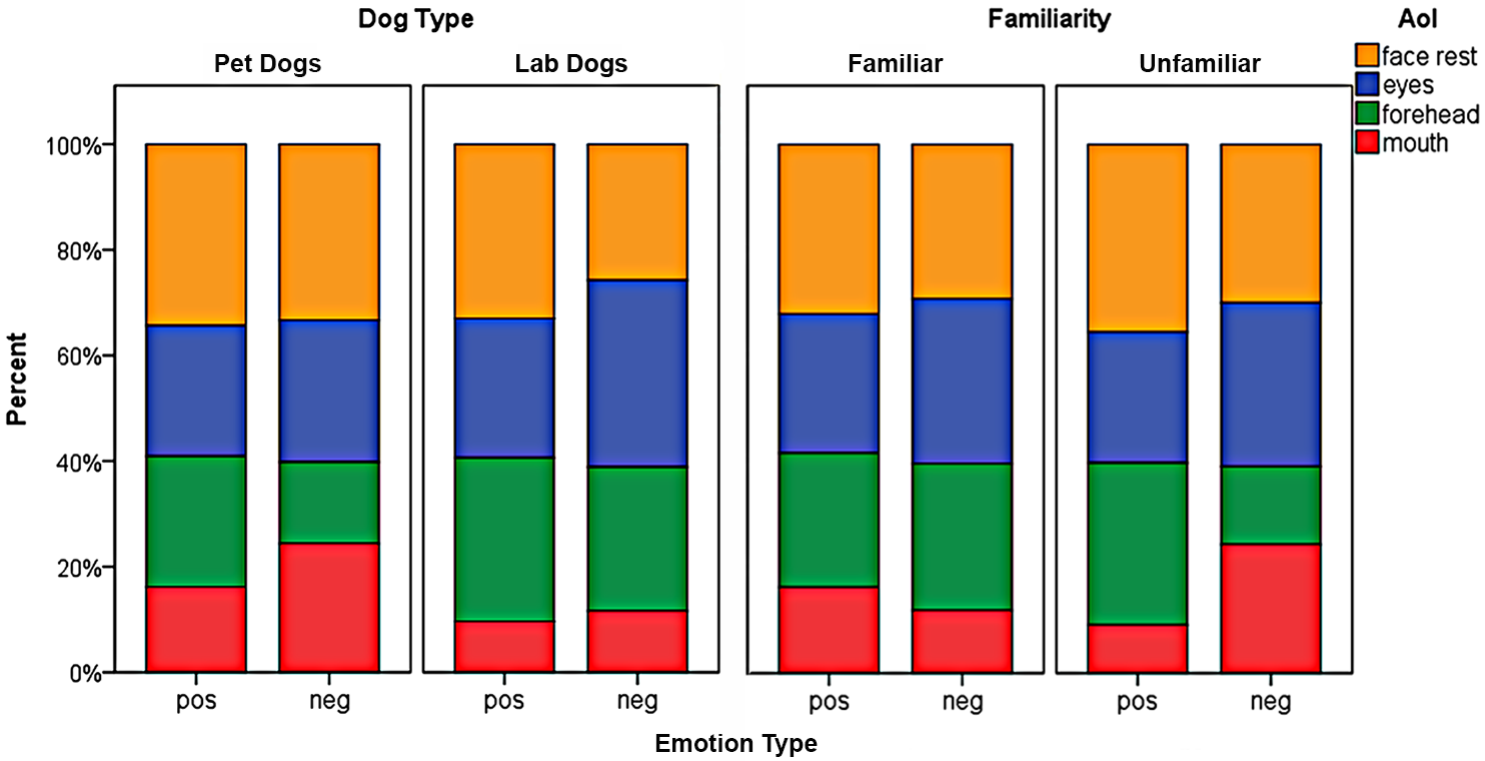
Frequencies [%] of the first fixations into the areas of interest subdivided for the emotion type with the subcategories dog type and familiarity.

Lab dogs looked more frequently into the in-face area with their first fixation (in 66% of all first fixations) than pet dogs (39%) (GLM LRT Chi²_1_ = 114.6, p<0.001; see also Table 3). The two dog types differed in the location of the face in which they looked first (multinomial GLMM F_3,779_ = 3.54, p = 0.014) and this measure was also influenced by the emotion type (F_3,779_ = 2.84, p = 0.037) and the familiarity (F_3,779_ = 6.54, p<0.001) of the stimulus. In comparison to the pet dogs, the lab dogs looked more likely first to the eyes or the forehead and less likely first to the mouth region (see Fig 7 left half). In addition, eyes and mouth were fixated first with a higher probability if the face expressed a negative emotion. The forehead and face rest were fixated first with a higher probability if the face expressed a positive emotion (see Fig 7). Further, there was a significant interaction between the emotion type and familiarity of the face (F_3,779_ = 6.54, p<0.001). For unfamiliar faces the probability to look first at the mouth was higher for negative than for positive expressions whereas the opposite pattern appeared for the familiar faces. Conversely, the probability to first fixate the forehead was higher for positive than for negative expressions when unfamiliar faces were shown but not when familiar faces were shown (see Fig 7 right half).

**Table 3:** Fixation count and probability of the first fixations into the face area (Face) and the rest of the picture (Picture) as well as the classification of fixations to the face into the areas of interest.

	Region of first fixation			First Area of Interest fixated				
Condition	Picture rest	In-Face	p	Eyes	Mouth	Forehead	Face Rest	p
Total								
Count	841	794		225	123	195	251	
%	51.4	48.6		28.3	15.5	24.6	31.6	
pet dog								
Count	636	399	**<0.001**	103	81	80	135	**0.014**
%	61.4	38.6		25.8	20.3	20.1	33.8	
lab dog								
Count	205	395		122	42	115	116	
%	34.2	65.8		30.9	10.6	29.1	29.4	
familiar								
Count	406	411	0.151	118	58	109	126	**<0.001**
%	49.7	50.3		28.7	14.1	26.5	30.7	
unfamiliar								
Count	435	383		107	65	86	125	
%	53.2	46.8		27.9	17.0	22.5	32.6	
positive								
Count	444	395	0.262	101	51	110	133	**0.037**
%	52.9	47.1		25.6	12.9	27.8	33.7	
negative								
Count	397	399		124	72	85	118	
%	49.9	50.1		31.1	18.0	21.3	29.6	

### Latency of the first fixation into the face

**Fig 8:**
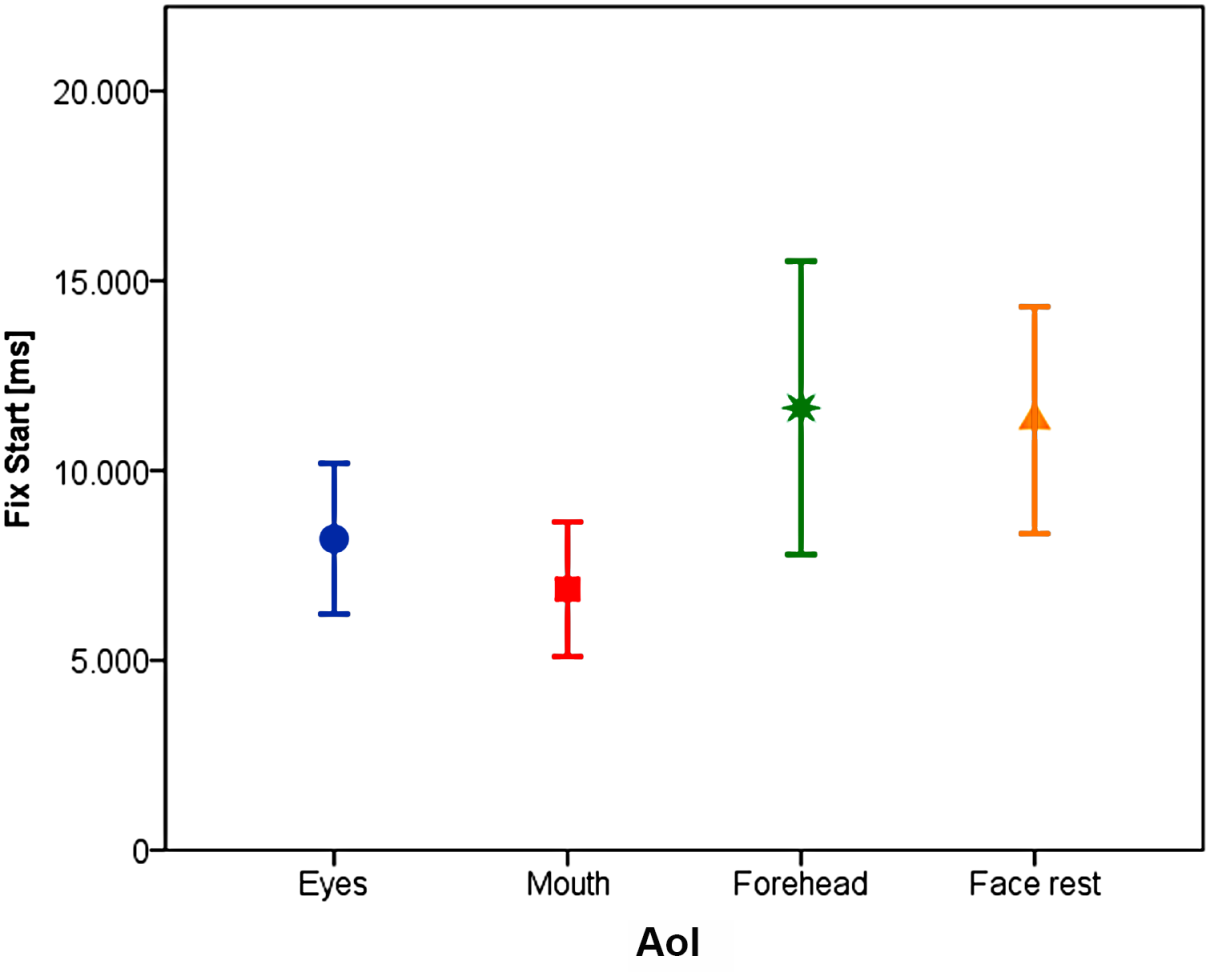
Mean fixation start (+/- 95% CI) of the first fixation directed into the areas of interest.

On average, the lab dogs fixated into the in-face area with the first fixation (1.05 +/- 0.02), whereas the pet dogs needed on average 1.5 +/- 0.09 fixations to do so. This difference was highly significant (GLMM F_1,-398_ = 15.42, p<0.001), but there was no influence of emotion type (F_1,-397.3_ = 3.23, p = 0.07), familiarity (F_1,-397.3_ = 1.03, p = 0.31) or area of interest (F_1,-397.3_ = 3.68, p = 0.3). Even though the pet dogs needed on average 1.5 fixations to fixate the in-face area they still looked sooner into the in-face area (on average after 8049 +/- 623ms) compared to lab dogs (on average after 11635+/- 1368ms). This difference was significant (LMM: F_1,103.5_ = 5.10, p = 0.026), but there was no influence of emotion type (F_1,756.3_ = 1.492, p = 0.222) or familiarity of the face (F_1,746.8_ = 0.901, p = 0.343).). If the first fixation into one of the four in-face AoIs went to the eyes or the mouth region, the latency to the first fixation was significantly shorter than if the first fixation went to the forehead or the face rest region (eyes: 8203 +/- 1007, mouth: 6875.7 +/- 896 vs. forehead: 11652.4 +/- 1960, face rest: 11330.9 +/- 1516; F_3,779.003_ = 3.892, p = 0.009, see Fig 8).

### Gaze Bias

**Fig 9:**
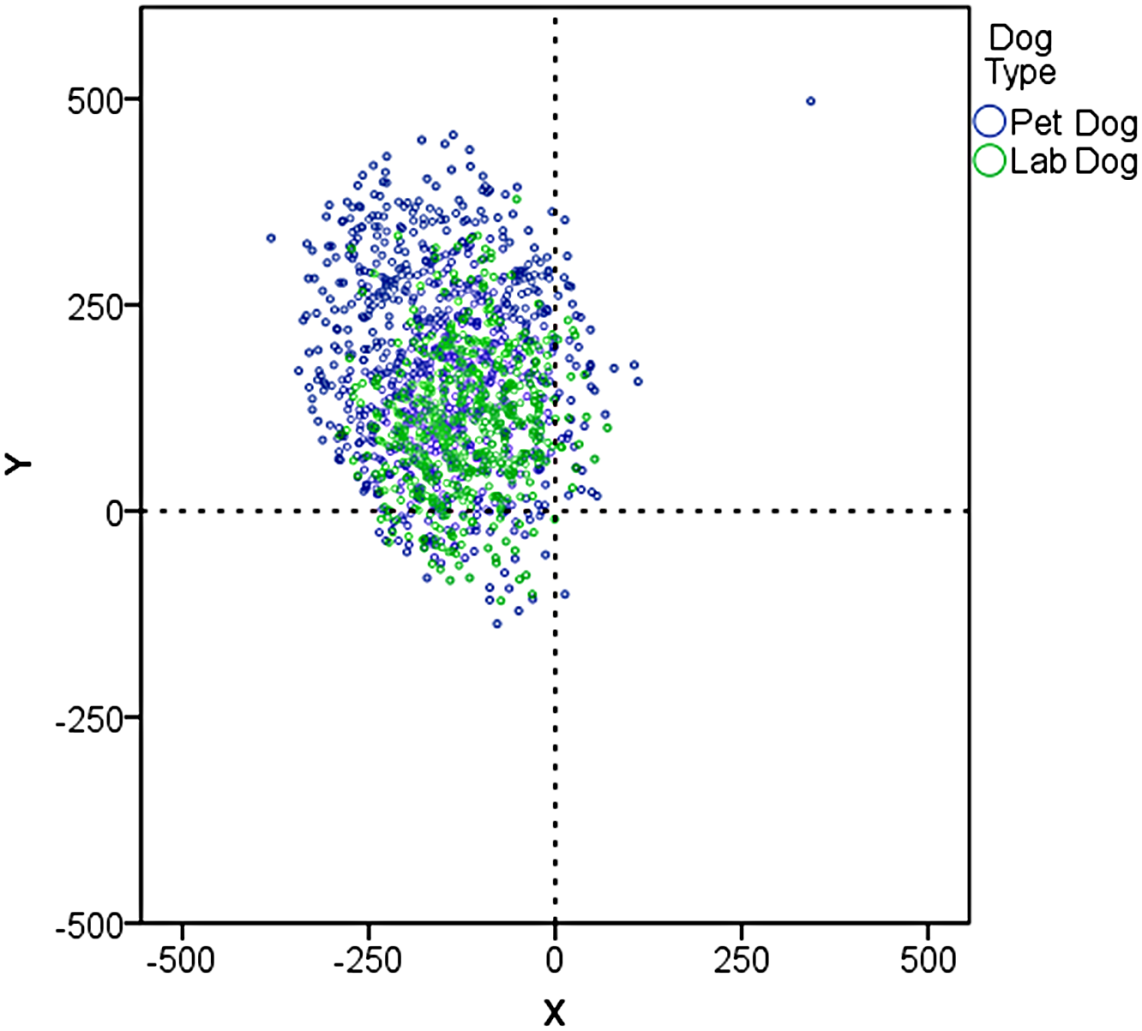
Coordinates of the first fixation made into the face region subdivided for the dog type. The origin corresponds to the middle of the vertical and horizontal dimension of the face.

The dogs showed a significant left gaze bias for the first fixation into the face (GLMM: estimate = 2.69 +/- 0.19, z = 14.12, p<0.001), with 92,5% of the first fixations being directed towards the right side of the face (in the dog's left visual field, see Fig 9). There was no significant difference between the dog types (estimate = -0.48 +/- 0.37, z = -1.29, p = 0.2). Additionally the gaze bias was neither influenced by the familiarity (estimate = -0.14 +/- 0.21, z = -0.66, p = 0.5) nor the emotion type (estimate = -0.04 +/- 0.2, z = -0.18, p = 0.9). Also the analysis of the fixation duration of all the fixations directed into the face region showed a significant preference for the left visual field (estimate = 4.62 +/- 0.45, z = 10.27, p<0.001). On average, 75% of the fixation dwell time was measured on the right side of the face. Also for this variable, there was no significant effect of emotion type (estimate = 0.16 +/- 0.45, z = 0.36, p = 0.7), familiarity (estimate = -0.37 +/- 0.45, z = -0.82, p = 0.4) or dog type (estimate = -0.49 +/- 0.68, z = -0.72, p = 0.4), but a tendency for an interaction between dog type and familiarity (estimate = -1.99 +/- 1, z = -1.95, p = 0.05). Lab dogs (estimate = -1.5 +/- 0.8, z = -1.88, p = 0.06) but not pet dogs (estimate = 0.48 +/- 0.64, z = 0.75, p = 0.5), tended to show a more pronounced left gaze bias towards familiar faces compared to unfamiliar faces.

## Discussion

In a nutshell, the findings of this study confirm all three major expectations about the processing of human faces by dogs: (1) an influence of the amount of exposure to humans and/or the quality of the human-dog relationship, as exemplified by the difference between pet and lab dogs; (2) (subtle) influences of both the familiarity and the emotional expression of the face; (3) a strong left gaze bias. Although in general these findings are in line with earlier studies on dogs' looking patterns towards human faces, there are some interesting deviations and a few unexpected results.

Concerning the looking differences between pet and lab dogs the former looked much earlier into the face region than the later. Possibly because (our) lab dogs live in packs and are therefore surrounded by other dogs but not humans, human faces are not salient enough to elicit a fast response. However, their very first fixation into the in-face region and the total looking time were longer compared to pet dog subjects. As a human face and especially human emotional expressions are not part of their daily visual environment, it is plausible that it takes lab dogs longer to process the facial expression. This is in line with the hypothesis of face-expertise, according to which face processing is facilitated by experience or familiarity with the stimulus [22,73–75]. A shorter viewing time might indicate faster and therefore more experienced processing. An alternative explanation for the increased looking behavior of lab dogs is perceptual preference for the faces in general and some face regions, such as the forehead or eye region, in particular. Further tests with familiar and unfamiliar as well as preferred and non-preferred stimuli would be necessary to rule out these differences between the pet and lab dogs. Another interesting difference between the dog types has been uncovered in the analysis of the location of the first fixation. While lab dogs fixate firstly the eye and forehead region and later the mouth region, especially if the face shows a negative emotion, pet dogs allocate their first fixation equally into the eyes, the forehead and the mouth region. This result indicates that, besides the importance of the eye region, the mouth region is of very high importance for the pet dogs, especially if the face shows a negative emotion. It seems plausible that pet dogs are tuned to pay more attention to the region of the mouth as they are expecting verbal commands from their owners on a daily basis. However, we cannot exclude the possibility that these differences emerge from asymmetry in the distribution of breeds across the lab and pet dogs. It has been postulated that pet dogs, which emerge from working breeds, are tuned to human communicative signals. Further they outperform dogs of non-working breeds, which are not breed to cooperate, and ancient dog breeds, which additionally had limited access to humans [76]. Hence, it is plausible that dogs with an extended access to humans underlie an additional selection on cooperative behavior. However, when adding breed group as a factor to the statistical models, we could not find a significant difference between the groups of herding, hunting and protection dogs in comparison to the lab dogs, which are originally hunting dogs as well. Still, because of the small sample sizes of the different breed groups we cannot entirely exclude an effect of the breeds. Therefore it would be desirable for future studies to compare only homogeneous groups of dogs to control for possible influences of breed characteristics.

A slightly different picture emerges, if we consider the latency to fixate the different face regions. Here both dog types showed a similar pattern, namely a much shorter latency for the eyes and mouth region than for the forehead or the remaining parts of the face. If we assume this measure as being indicative of the dogs' primary interest for eyes and mouth, it would conform with previous investigations on humans and dogs [63,64,77,78]. The eyes and the mouth of the human face have been assumed being the most informative parts as they provide the primary communicative cues for human-dog communication.

Concerning the influence of the emotional expression of the human face on the dog's looking patterns we got mixed results. On the one hand, the data confirm an influence, as the fixation count was significantly higher for the forehead if a positive expression was displayed but higher for the mouth and eye region if a negative expression was displayed. On the other hand, the data are difficult to interpret, as they deviate from what is known from the literature. In humans the mouth in positive emotions and the eyes in negative emotions are receiving most attention [79] and these regions display the most characteristic changes during positive and negative emotional expressions, respectively (pulled up lip corners or lower eye lids, [80]). In the current study, the mouth received not much attention when dogs looked at faces with positive emotions, at least when using first fixation data. When looking at faces displaying negative emotions the eyes received much more attention than the mouth, especially in lab dogs, which correspond with the data from humans. With our scientific questions and resulting set up, it might be a possible weakness that we were not able to use human emotional expressions of validated data-bases. Instead we were using pictures of individuals that were not trained to express emotions in the face. This resulted in pictures that may have been ambiguous to some observers, and possibly also in ambiguous findings on the influence of the emotion on the face processing. Further investigations, particularly of prototypical faces with stimulus manipulations of the valence of the expressed emotions or a mixture of facial parts of different emotional expressions (e.g. “happy forehead” with “angry mouth”), would help clarifying these ambiguous findings.

A somewhat similar ambiguity emerged for interpreting the data indicating an influence of familiarity. Here again the influence is visible only in the first fixations. If unfamiliar faces but not familiar ones with a negative expression were shown dogs looked preferentially to the lower face region in their first fixation. We may therefore suggest a gaze aversion effect known from humans studies; adult humans exhibit a gaze aversion when confronted with threatening stimuli [81]. The fact that the dog subjects of our study showed this effect only when confronted with unfamiliar faces may be explained by their inability to retrieve positive associations with these faces. Therefore they may perceive this unfamiliar faces with negative expressions as not “trustworthy” or even threatening [64] and avoid the eye contact. Overall, unlike suggested by the findings of other studies [63,64,66], we could not find a clear preference for familiar faces, as they received the same number of fixations and were scanned for the same duration as unfamiliar faces. This result is surprising concerning the theory of face expertise which postulates that familiarity with a stimulus results in less and shorter fixation [18,19]. It is possible that the differences between the familiar and unfamiliar faces are too marginal or that due to a repeated exposure to the same stimuli the effect of familiarity was overwritten. Further investigations on stimuli processing of familiar and unfamiliar stimuli will be necessary to disentangle this result.

The strongest support for our expectations comes from the analysis of the lateral eye movements, resulting in looking into the left and right halves of the face. The data revealed a strong left gaze bias, i.e. looking preferentially into the right face hemisphere in the left visual field of the dog. Such a preference for the left visual field is associated with the engagement of the opposite, here the right brain hemisphere. This finding is not only very robust but in general also corroborates the results of previous studies [43,44]. In both studies dogs displayed a left gaze bias when viewing human faces with neutral expressions. In addition, a left gaze bias was also observed towards negative facial expressions, but not towards positive expressions [43]. The later results are somehow at odds with our data. We found no variations in their gaze bias between the four different emotional expressions. The dogs in this study showed the same strong bias also to faces with a positive emotional expression. However, the absence of a left gaze bias towards positive expressions in Racca et al. [43] may be due to their measuring of the gaze over an extended time period rather than the initial onset and by their methodological reliance on the analysis of video-based gaze directions. Our measurements are in line with studies on humans showing that the gaze bias towards emotive stimuli has a very early onset [44,82,83] and are based on the much more precise and objective eye-tracking method.

In general, our data are more in line with the Right Hemisphere Model [46], suggesting the regulation of emotional processes by the right hemisphere regardless of their valence, than the Valence Model [48], suggesting a left gaze bias only towards negative emotions. This result corresponds to findings on a variety of species, which show a lateralization towards emotive stimuli regardless of the valence (e.g. [51–55,59]). As 'neutral' human faces, i.e., faces showing no facial muscle contraction, may be perceived as negative (cold or threatening) by humans [84], the strong left gaze bias also to 'neutral' faces does not weaken our conclusion that the dog subjects perceived the human faces as emotional. Lesion studies on human subjects showed that if the processing of the right hemisphere is disturbed, subjects are not able to recognize emotions [85,86]. Therefore it is likely that dogs, due to the significant left gaze bias in this study and their ability to discriminate human facial expressions [69], are indeed able to recognize emotional expressions in humans. However, further studies are necessary to clarify the nature of this *recognition*, i.e. which associations dogs have with different human emotions and which consequences they anticipate following their perception.

## Supporting Information

S1 Table Complete dataset. (XLSX)
